# Multi-Modal and Molecular Imaging of Cellular Microenvironment and Tissue Development

**DOI:** 10.3390/ijms23137113

**Published:** 2022-06-26

**Authors:** Francesco Pampaloni

**Affiliations:** Physical Biology Group, Buchmann Institute for Molecular Life Sciences (BMLS), Goethe-Universität Frankfurt am Main, Max-von-Laue Str. 15, 60438 Frankfurt, Germany; fpampalo@bio.uni-frankfurt.de

Imaging the interaction of individual cells with their surrounding tissue microenvironment is essential to advance in bioprinting, tissue engineering and cancer biology, to mention just three highly relevant fields in the life sciences. The microenvironment is essentially composed by the tissue-specific neighbouring cells (e.g., gut epithelial cells), fibroblasts, endothelial cells, immune cells, and the extra-cellular matrix (e.g., the adjacent basement membrane or the collagen type I-rich tissue stroma). During the last decade, a variety of new technologies for the imaging of large three-dimensional cell cultures and whole organs have been developed.

Imaging technologies with different contrast modalities, each having their individual advantages and drawbacks, are required to image these large and highly heterogeneous samples. Critically, the choice of a specific imaging technology depends on the particular tissue and the biological questions to address.

Fluorescence microscopy, by taking advantage of fluorescent fusion proteins and immunofluorescent tags, provides information about the spatial distribution and the temporal evolution of distinct cellular populations in a tissue (for instance, in processes such as the differentiation and migration of macrophages) and of their molecular interactions at intra-cellular level (e.g., the integrity of mitochondria or the formation of autophagosomes), inter-cellular level (e.g., the formation of desmosomes, EpCAM, tight junctions), and cell-matrix level (e.g., the establishment of focal adhesions). Linear (one-photon) fluorescence microscopy suffers from a limited penetration depth. This is only partially improved by the introduction of Light Sheet Fluorescence Microscopy (LSFM) [1] and by the use of fluorescent probes in the near infrared spectral range. Non-linear techniques, such as two-photon fluorescence microscopy, leverage better tissue penetration of infrared light excitation and provide a longer imaging depth (up to ~600 µm [2]) as well as the intrinsic optical sectioning made possible by the two-photon beam waist.

While the high selectivity and signal-to-noise ratio obtainable with fluorescence microscopy are outstanding, a clear drawback is the need of fluorescent probes, although endogeneous autofluorescence can also in some cases provide useful information.

Label-free microscopy is currently a highly active and pertinent field of research. Label-free microscopy comprises a broad variety of contrast methods that rely on different linear and non-linear physical and photophysical processes. A non-exhaustive list includes low-coherence interferometry (optical coherence tomography, OCT) [3], light phase shift (digital holographic microscopy, DHM) [4], Raman scattering (Raman microscopy and coherent anti-Stokes Raman scattering microscopy, CARS) [5], Brillouin scattering [6], second- and third-harmonic generation (SHG, THG), and photoacoustic microscopy (PAM) [7].

The obvious drawback compared to fluorescence microscopy and common to all label-free methods is the limited specificity of the signal. However, the combination of two (or possibly more) approaches in multi-modal systems, for example the combination of OCT with LSFM [3] and of OCT with PAM [8] allows taking advantage of the strengths of individual techniques and to effectively compensate for their intrinsic limitations.

Beside imaging based on the visible and near-IR spectral range, positron emission tomography (PET), micro-CT [9] and the most recent phase-contrast tomography [10] also allow the high-resolution imaging of in vivo and fixed specimens.

The contributors of this Special Issue present their latest research in these exciting areas, highlighting how multimodal imaging approaches provide a deep insight into the cellular processes and interactions in highly heterogenous tissue microenvironments. 

Rakhymzhan et al. [11] describe a novel spectral unmixing algorithm based on dimension reduction in diagonalized matrices. The algorithm allows improving multiplexed two-photon imaging of whole organs. Multiplexed two-photon laser scanning microscopy achieves deep imaging of live and intact tissue specimens, in contrast to cyclic immunofluorescence that is only suitable for fixed tissue slices.

Yang et al. [12] present a new miniaturized fluorescence molecular tomography (FMT) endoscope and demonstrate its application for in vivo imaging of breast cancer in mice. The authors used targeted iron oxide nanoparticles (IONPs) labeled with a near-IR fluorophore as a contrast agent.

Parodi et al. [13] show the application of non-invasive and label-free CARS and SHG microscopies to the phenotypic characterization of rat mesenchymal stem cells (MSC) cultured in a microfabricated porous scaffold termed “Nichoid”. The processes of adipogenic and chondrogenic differentiation of the MSC cultured inside the Nichoid substrate was studied with live TPEF/SGH microscopy over a time of up to 14 days, showing that this multi-modal approach provides more extensive datasets compared to confocal fluorescence microscopy.

With the aim of dissecting cell population heterogeneity in pediatric brain tumors Pericoli et al. [14] developed a method to generate fluorescent barcoded cell lines derived from primary pediatric glioblastoma and pontine glioma. The barcoding was achieved by random expression of six different fluorescent fusion proteins, an approach dubbed Multifluorescent Marking Technology. Two different imaging platforms (confocal microscope and Operetta CLS high-content/high-throughput) were used to validate the labeling technology in two 3D models of tumor invasion: invasion assays in Matrigel and in organotypic brain slices.

Nguyen et al. [8] integrate photoacoustic microscopy and optical coherence tomography in a novel multimodal imaging system. The authors used this technology to visualize and quantify experimental ischemic lesions in the eyes of live rabbits (choroidal vascular occlusion, CVO, induced by laser photocoagulation). The results show that high-resolution, high-contrast, label-free 3D volumetric imaging of CVO sites is achievable with the PAM/OCT system.

Finally, Valtorta et al. [15] provide a comprehensive overview of the inter- and intra-tumor heterogeneity in glioblastoma and its link with chemoresistance. The review focuses on the multimodal application of positron emission tomography (PET) and magnetic resonance imaging (MRI) for the in vivo imaging of glioblastoma. MR-PET, combined with selected radiopharmaceuticals, allows effective characterization of the biological features of glioblastoma (metabolism, hypoxia, inflammation) and its cellular microenviroment (e.g., endothelial cells, fibroblasts, astrocytes) and can help to better identify patient clusters sharing similar tumor phenotypes, which is essential to developing new chemo- and radiotherapeutic approaches.
